# Effect of withdrawal of thyroid hormones versus administration of recombinant human thyroid-stimulating hormone on renal function in thyroid cancer patients

**DOI:** 10.1038/s41598-023-27455-0

**Published:** 2023-01-05

**Authors:** Young-Sil An, Jeonghun Lee, Hyeung Kyoo Kim, Su Jin Lee, Joon-Kee Yoon

**Affiliations:** 1 Department of Nuclear Medicine and Molecular Imaging, Ajou University School of Medicine, Suwon, Korea; 2 Department of Surgery, Ajou University School of Medicine, Suwon, Korea

**Keywords:** Endocrinology, Risk factors

## Abstract

This study was conducted to investigate the effects of thyroid hormone withdrawal (THW) and recombinant human thyroid-stimulating hormone (rhTSH) administration on renal function in patients with thyroid cancer after total thyroidectomy. This study included 202 patients who discontinued thyroid hormone therapy and/or received rhTSH after total thyroidectomy. Creatinine (Cr), blood urea nitrogen (BUN) levels, and estimated glomerular filtration rate (eGFR) were assessed at the following three time points: before thyroidectomy, at least 3 weeks after THW, and 1 day after the second injection of rhTSH. The median serum Cr level was significantly higher following THW compared to that before thyroidectomy (0.95 versus 0.70). In contrast, the median BUN level was significantly lower after THW compared to that before thyroidectomy (9.8 versus 11.3). Over a fifth (22.2%) of patients had abnormal eGFR values after THW, which was significantly greater than that before thyroidectomy. In contrast, renal parameter values after rhTSH administration were not significantly different than those before thyroidectomy. In conclusion, THW affects renal function in patients with thyroid cancer who have undergone total thyroidectomy. However, renal function in such patients is not affected by rhTSH administration.

## Introduction

Radioactive iodine (RAI) therapy is frequently performed after total thyroidectomy in patients with differentiated thyroid cancer, as it can ablate residual thyroid tissue. RAI can also be used to treat metastatic lesions in the lymph nodes, lungs, and bones^1,2^. In order to facilitate effective RAI therapy, thyroid-stimulating hormone (TSH) levels should be raised to at least 30 mU/L; this requires that patients stop taking thyroid hormone pills for at least 3 weeks^3^. Thyroid hormones are known to affect kidney structure, glomerular filtration rate (GFR), and renal hemodynamics either directly through renal actions or by mediating systemic hemodynamic effects^4^. Several studies have reported that hypothyroidism adversely affects renal function^5–8^. Therefore, if patients with thyroid hormone withdrawal (THW) develop hypothyroidism, they are also presumed to be at a greater risk of experiencing adverse effects on kidney function. However, only a limited number of studies to date have evaluated the effect of the discontinuation of thyroid hormone medication on renal function^9–13^.

Recombinant human TSH (rhTSH) injection is widely used in place of THW to facilitate RAI treatment in patients with thyroid cancer^14^. Indeed, rhTSH administration may potentially prevent hypothyroidism and adverse effects on renal function, as patients are still able to receive thyroid hormone medication; nevertheless, this has only been supported by a few studies^9–11^. Moreover, a previous study has also reported that rhTSH decreases renal perfusion and function^15^. There is currently no definite consensus on the renal effects of rhTSH. Therefore, the objective of this study was to evaluate and compare the effects of rhTSH administration and THW on renal function in patients undergoing total thyroidectomy.

## Results

### Patient characteristics

The median age of the 202 patients included in this study was 42 (interquartile range [IQR] 35–50) years, and women accounted for 67.3% (136/202) of the patients. The most common pathologic type of thyroid cancer was papillary carcinoma (197/202, 97.5%), and 85.1% of the patients (172/202) were in American Joint Committee on Cancer (AJCC) stage I. Of the 202 patients, 176 discontinued thyroid hormone therapy and 26 were administered rhTSH. The detailed clinical characteristics of the patients included in this study are described in Table 1.

**Table 1:** Patient characteristics.

	Thyroid hormone withdrawal (*n* = 176)	Administration of rhTSH (*n* = 26)	Total (*n* = 202)
Age, median (IQR), years	43 (35–50)	41 (33–46)	42 (35–50)
**Sex, n (%)**			
Female/male	117 (66.5%)/59 (33.5%)	19 (73.1%)/7 (26.9%)	136 (67.3%)/66 (32.7%)
**Pathologic type, n (%)**			
Papillary carcinoma/follicular carcinoma	173 (98.3%)/3 (1.7%)	24 (92.3%)/2 (7.7%)	197 (97.5%)/5 (2.5%)
**AJCC stage, n (%)**			
I/II/III/IV	149 (84.7%)/18 (10.2%)/2 (1.1%)/7 (3.9%)	23 (88.5%)/2 (7.7%)/0 (0%)/1 (3.8%)	172 (85.1%)/20 (9.9%)/2 (1.0%)/8 (4.0%)

*rhTSH* recombinant human thyroid-stimulating hormone, *IQR* interquartile range, *AJCC* American Joint Committee on Cancer.

### Effects of THW on renal function

**Figure 1:**
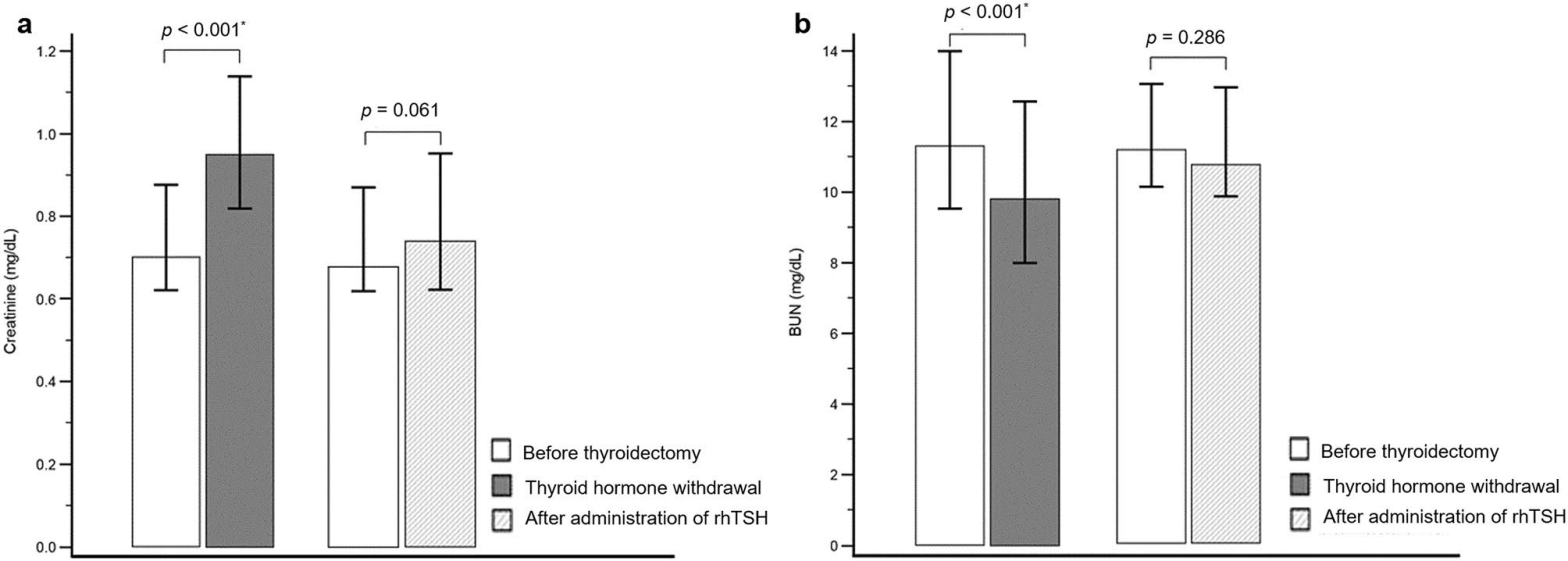
Effects of THW or rhTSH administration on renal parameters. (**a**) The Cr level was significantly higher after THW than before thyroidectomy (*p* < 0.001). However, there was no significant difference in the Cr level after rhTSH administration compared to that before thyroidectomy (*p* = 0.061). (**b**) When thyroid hormone therapy was discontinued, BUN was significantly decreased from levels observed prior to thyroidectomy (*p* < 0.001). The administration of rhTSH did not affect the BUN level (*p* = 0.286). *BUN* blood urea nitrogen, *Cr* creatinine, *rhTSH* recombinant human thyroid-stimulating hormone, *THW* thyroid hormone withdrawal.

The median serum Creatinine (Cr) level was significantly higher following THW compared to that before thyroidectomy (0.95 [IQR 0.82–1.14] versus 0.70 [IQR 0.62–0.88], *p* < 0.001; Fig. 1a). In contrast, the median blood urea nitrogen (BUN) level was significantly decreased after THW compared to that before thyroidectomy (9.8 [IQR 8.0–12.6] versus 11.3 [IQR 9.5–14.0], *p* < 0.001; Fig. 1b).

Among patients who underwent THW, all exhibited a normal estimated GFR (eGFR) (> 60 mL/min/1.73 m^2^) prior to total thyroidectomy. After THW, 22.2% (43/194) had abnormal eGFR values, which was significantly different from that recorded before total thyroidectomy (*p* < 0.001).

### Effects of rhTSH on renal function

The median serum Cr level following rhTSH administration was not significantly different than that prior to thyroidectomy (0.74 [IQR 0.62–0.95] versus 0.68 [IQR 0.62–0.87], *p* = 0.061; Fig. 1a). Similarly, the median BUN level before thyroidectomy was not significantly affected by rhTSH administration (11.1 [IQR 10.1–13.0] versus 10.7 [IQR 9.8–12.9], *p* = 0.286; Fig. 1b). The eGFR was > 60 mL/min/1.73 m^2^ in all patients both before thyroidectomy and after rhTSH administration; therefore, statistical analysis was not possible.

### Comparison of renal effects of THW and rhTSH administration

**Figure 2:**
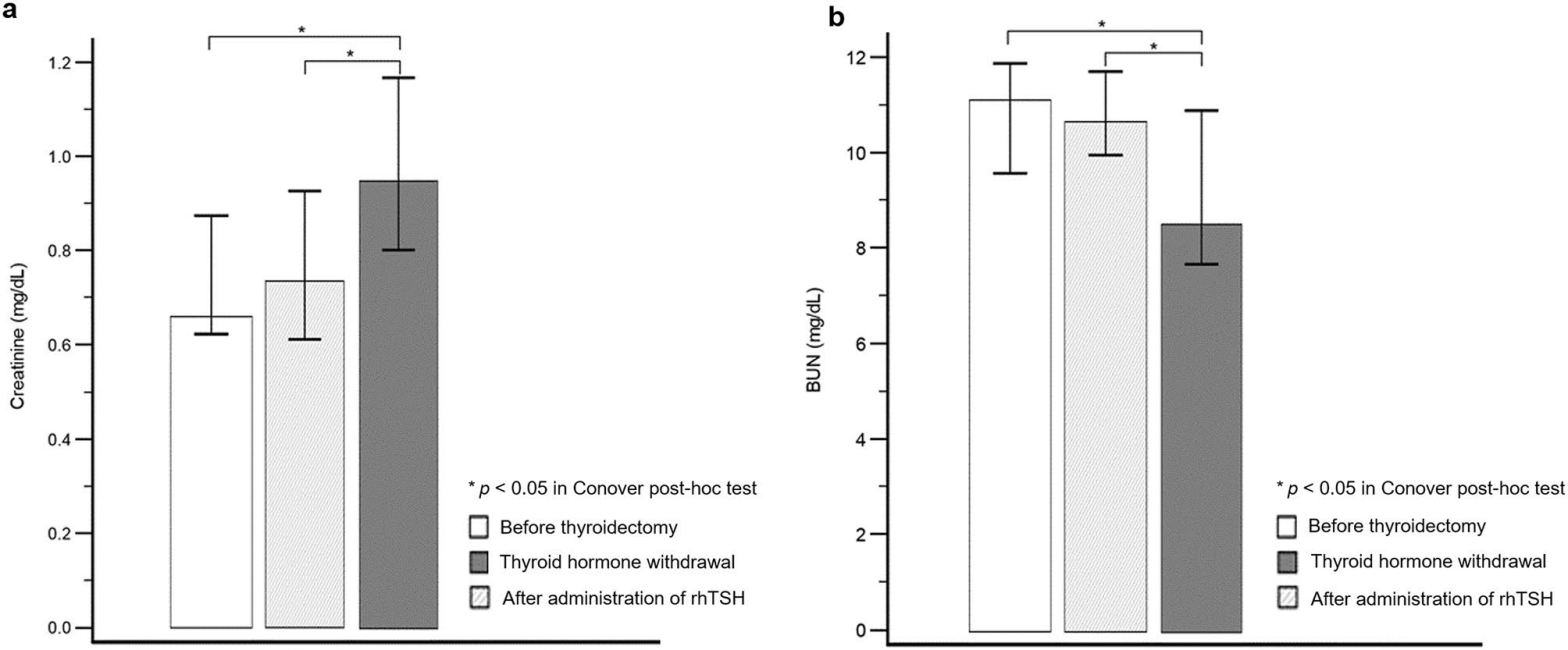
Comparison of renal parameters under each condition in the same patient group. (**a**) The Cr level was significantly higher after THW compared to that following rhTSH administration and before thyroidectomy (both *p* < 0.05 in Conover post-hoc test). However, the Cr level after rhTSH administration was not significantly different compared to that before thyroidectomy. (**b**) BUN after THW was significantly lower than that after rhTSH administration and before thyroidectomy (both *p* < 0.05 in Conover post-hoc test). *BUN* blood urea nitrogen, *Cr* creatinine, *rhTSH* recombinant human thyroid-stimulating hormone, *THW* thyroid hormone withdrawal.

Eighteen patients were both administered rhTSH for RAI therapy and underwent THW for follow-up 6 months after therapy. This facilitated within-patient comparisons of renal parameters before thyroidectomy and after THW and rhTSH administration. Cr levels were significantly different under these three conditions (*p* < 0.001). The median Cr level following THW (0.95 [IQR 0.78–1.23]) was significantly greater than that after rhTSH administration (0.74 [IQR 0.60–0.99], *p* < 0.05; Fig. 2a) and before thyroidectomy (0.66 [IQR 0.62–0.90], *p* < 0.05; Fig. 2a). However, the median Cr level after rhTSH administration was not significantly different than that before thyroidectomy (*p* > 0.05).

BUN levels were also significantly different under the three conditions (*p* = 0.029). However, unlike the trend observed with Cr, the lowest median BUN level was observed after THW (8.5 [IQR 7.6–11.1]); this was significantly lower than that after rhTSH administration (10.7 [IQR 9.8–11.9], *p* < 0.05; Fig. 2b) and before thyroidectomy (11.2 [IQR 9.3–12.0], *p* < 0.05; Fig. 2b). The median BUN level after rhTSH administration was not significantly different from that before thyroidectomy (*p* > 0.05).

Following the discontinuation of thyroid hormone therapy, only a single patient exhibited an abnormal eGFR. All patients with other conditions (after rhTSH administration and before thyroidectomy) showed normal eGFR. They failed to show a statistically significant difference (*p* = 0.368).

## Discussion

Hypothyroidism may develop when patients discontinue thyroid hormone therapy that was originally administered to stimulate TSH after total thyroidectomy because of thyroid cancer. Hypothyroidism is associated with a wide array of symptoms, including weight gain, constipation, fatigue, and puffy face. Changes in kidney function may also be evident^16,17^. As thyroid and renal function are closely related, some studies have recommended the routine assessment of GFR based on serum Cr levels in patients with thyroid disorders^18^. For this reason, our institution routinely monitors renal function parameters in patients undergoing THW or rhTSH treatment.

The results of the present study indicated that THW significantly increased serum Cr and decreased eGFR to an abnormal level, thus reflecting adverse effects on the kidney. These results are consistent with those of previous studies^8,9,12,13,17^. It is known that renal function is decreased in hypothyroidism owing to an increased serum Cr level^19^. The decrease in GFR during a hypothyroid state is thought to be due to the direct or indirect effects of cardiovascular and systemic hemodynamic factors^4^. The results of our study highlight the adverse effects of THW-induced hypothyroidism on renal function. While temporary THW-associated impairments in renal function can be managed with thyroid hormone replacement in the majority of cases^6,8,10,13^, their impact on patient health remains unknown. This warrant increased attention and consideration by clinicians.

Serum BUN level was significantly decreased by THW in the present study. Since BUN is used as a clinical indicator of renal function, in conjunction with Cr^20^, we expected that levels would increase following THW, similarly to Cr. However, an opposite trend was observed. There is a lack of studies that have evaluated BUN levels in patients with hypothyroidism. Contrary to our results, Kumari et al.^21^ reported that hypothyroidism raised BUN levels relative to a normal control; this was associated with a derangement of renal function^21^. These discrepant results may possibly be due to differences in sample size, which was larger in our study (*n* = 176 versus *n* = 35). The decrease in BUN level after THW in our study could be attributed to a decrease in liver function caused by hypothyroidism. Indeed, the BUN level reflects the balance of urea production in the liver and urea elimination in the kidney. It therefore may be used as an indicator of the status of both organs^20,22^. A number of previous studies have reported that hypothyroidism alters liver function^9,23–26^. In particular, Lee et al.^9^ observed that aspartate transaminase and alanine transferase were elevated by THW in patients undergoing RAI for thyroid cancer. Nevertheless, as we were unable to evaluate liver function parameters in the present study, a definitive relationship with BUN levels could not be confirmed. Moreover, BUN levels can be affected by diet or various physiologic conditions, regardless of liver or renal function^20^. Additional studies are required to address the specific cause of the decrease in BUN levels after THW.

The results of the present study indicated that rhTSH did not have an effect on serum Cr or GFR, which is consistent with results of most previous studies^9–11^. For example, Coura-Filho et al. reported that GFR impairment was due to hypothyroidism caused by THW, as opposed to rhTSH administration and TSH stimulation^11^. In addition, rhTSH did not have any effect on BUN levels in our study; this is supported by previous studies that have reported a lack of change in liver function after rhTSH administration^9^. However, Saracyn et al.^15^ found that rhTSH significantly reduced renal cortical perfusion and GFR; this was attributed to the ability of rhTSH to constrict small renal vessels by acting on receptors in their smooth muscle cells. These contrasting results may be because of the fact that Saracyn et al.^15^ measured basal renal parameters immediately before rhTSH administration, whereas we recorded these parameters before total thyroidectomy. In fact, we have an average interval of about 4 months between the baseline and renal function values after rhTSH administration, which may be a relatively weak point compared to the study of Saracyn et al.^15^. It seems difficult to conclude here whether any of the results reported by us and Saracyn et al.^15^ are correct. However, there are still more reports that rhTSH has no effect on renal function, and we would like to say here that our present findings further support this.

A strength of our study was that the renal effects of THW and rhTSH were evaluated in the same patients, although the number of patients was relatively small (*n* = 18). Comparing renal parameters under the three different conditions (prior to thyroidectomy and after THW and rhTSH administration) in the same patient has the advantage of nullifying the confounding effects of other clinical variables that may differ between patients. The results also showed that THW caused a significant increase in Cr levels, whereas rhTSH did not, proving once again that THW affects renal function. As such, that hypothyroidism caused by THW has a harmful effect on kidney was clearly shown through various analysis of our study, and we also thought about how to apply it clinically. We cautiously recommend that rhTSH should be administered as a substitute for THW if the clinician is concerned that the patient's temporary decrease in renal function may have adverse clinical effects. Moreover, the clinician should be more cautious in patients with already impaired baseline renal function.

This study had several limitations. First, as we wanted to solely evaluate the effect of THW and rhTSH administration on renal function parameters, we did not include patients with underlying kidney disease or a medical history of conditions that could cause abnormal kidney function. Further studies will be needed to determine whether our results in patients with a healthy baseline renal function are applicable to patients with an existing renal function impairment. Second, the number of patients receiving rhTSH in our study (*n* = 26) was relatively small compared to the number of patients (*n* = 176) with THW. This may have been caused by a lower patient preference for rhTSH administration owing to the additional costs. Nevertheless, we verified that this sample size was sufficient to facilitate a reliable statistical analysis. Third, our retrospective study does not include long-term follow-up of renal parameters of patients. This study only revealed the short-term effects of THW and rhTSH administration on the kidney, not their long-term effects. Although previous studies have reported that most of the deterioration of renal function caused by THW can be recovered by taking thyroid hormone^6,8,10,13^, more studies on their long-term or permanent effects are needed. Finally, the only renal function parameters evaluated were serum BUN, Cr, and Cr-based eGFR. Notably, no quantitative changes in eGFR were observed, as we were only able to differentiate normal and abnormal levels based on a cutoff value of 60 mL/min/1.73 m^2^. While previous studies have evaluated renal function and perfusion by using Cr-51 ethylenediaminetetraacetic acid or Doppler ultrasonography to measure GFR^11,15^, we were unable to investigate these parameters owing to our use of a retrospective study design. Future prospective studies should be designed to evaluate a wider range of renal parameters.

In conclusion, THW affects renal function in patients with thyroid cancer who have undergone total thyroidectomy. In contrast, renal function in such patients is not affected by rhTSH administration.

## Methods

### Patients

This retrospective study enrolled a total of 264 patients with thyroid cancer who discontinued thyroid hormone therapy and/or received rhTSH for radioiodine treatment and were followed up at our hospital from May 2020 to November 2021. Among these patients, 62 with a past medical history of conditions (diabetes mellitus, 25 patients; hypertension, 35 patients; unilateral nephrectomy for renal cancer, 1 patient; end-stage renal disease, 1 patient) that could affect renal function were excluded. Therefore, 202 patients were finally included in the study.

A chart review was performed to record renal function parameters including Cr, BUN, and eGFR measured via blood tests before total thyroidectomy, at least 3 weeks after THW, and 1 day after the second administration of rhTSH. This study was approved by the appropriate institutional review board, and the requirement for informed consent was waived.

### Renal function parameters

The following reference intervals for renal function parameters were used: BUN (8.0–23.0 mg/dL) and Cr (0.50–0.90 mg/dL). The eGFR was calculated using the Modification of Diet in Renal Disease equation^27^, and a value of 60 mL/min/1.73 m^2^ or higher was considered normal.

### Statistical analysis

All statistical analyses were performed using MedCalc software (version 20.106; MedCalc Software Ltd, Ostend, Belgium). The required sample size was calculated based on a significance (α) level of 5% and statistical power (1 − β) of 80%. A sample size of 12 was required to obtain an appropriate confidence level F for the paired samples *t* test; thus, the smallest sample size finally achieved (*n* = 18) in this study satisfies this.

The Kolmogorov–Smirnov test was used to verify whether the continuous variables had a normal distribution. As all data were not normally distributed, values are expressed as median and IQR, and non-parametric statistical analyses were performed. The Wilcoxon test for paired samples and the Friedman test were used to analyze whether there was a statistically significant difference in BUN and Cr values before thyroidectomy, after THW, and after rhTSH administration. If there was a significant difference in the Friedman test, a pairwise comparison of variables was conducted according to the method described by Conover^28^. The McNemar test and Cochran *Q* test were used to determine whether eGFR changes were statistically significant in the THW and rhTSH groups. *p*-values < 0.05 were considered significant.

### Ethics declarations

This retrospective study was conducted in accordance to the guidelines of the Declaration of Helsinki and approved by the Institutional Review Board of Ajou University (AJOUIRB-MDB-2021-703), through which informed consent was waived.

## Data Availability

The datasets used and/or analyzed during the current study are available from the corresponding author on reasonable request.

## References

[1] Bal CS, Padhy AK (2015). Radioiodine remnant ablation: A critical review. World J. Nucl. Med..

[2] Verburg FA, Hanscheid H, Luster M (2017). Radioactive iodine (RAI) therapy for metastatic differentiated thyroid cancer. Best Pract. Res. Clin. Endocrinol. Metab..

[3] Luster M (2008). Guidelines for radioiodine therapy of differentiated thyroid cancer. Eur. J. Nucl. Med. Mol. Imaging.

[4] Mariani LH, Berns JS (2012). The renal manifestations of thyroid disease. J. Am. Soc. Nephrol..

[5] Mooraki A, Broumand B, Neekdoost F, Amirmokri P, Bastani B (2003). Reversible acute renal failure associated with hypothyroidism: Report of four cases with a brief review of literature. Nephrology (Carlton).

[6] den Hollander JG, Wulkan RW, Mantel MJ, Berghout A (2005). Correlation between severity of thyroid dysfunction and renal function. Clin. Endocrinol. (Oxf.).

[7] Chonchol M (2008). Prevalence of subclinical hypothyroidism in patients with chronic kidney disease. Clin. J. Am. Soc. Nephrol..

[8] Kreisman SH, Hennessey JV (1999). Consistent reversible elevations of serum creatinine levels in severe hypothyroidism. Arch. Intern. Med..

[9] Lee SJ, Lee HY, Lee WW, Kim SE (2014). The effect of recombinant human thyroid stimulating hormone on sustaining liver and renal function in thyroid cancer patients during radioactive iodine therapy. Nucl. Med. Commun..

[10] Cho YY (2019). Long-term outcomes of renal function after radioactive iodine therapy for thyroid cancer according to preparation method: Thyroid hormone withdrawal vs. recombinant human thyrotropin. Endocrine.

[11] Coura-Filho GB, Willegaignon J, Buchpiguel CA, Sapienza MT (2015). Effects of thyroid hormone withdrawal and recombinant human thyrotropin on glomerular filtration rate during radioiodine therapy for well-differentiated thyroid cancer. Thyroid.

[12] Massolt ET (2017). Effects of thyroid hormone on urinary concentrating ability. Eur. Thyroid J..

[13] van Velzen DM, Krul-Poel YH, den Heijer M, Simsek S (2018). Subacute renal injury in hypothyroidism: A case report of an unusual phenomenon. Neth. J. Med..

[14] Ma C (2010). Recombinant human thyrotropin (rhTSH) aided radioiodine treatment for residual or metastatic differentiated thyroid cancer. Cochrane Database Syst. Rev..

[15] Saracyn M (2020). Recombinant human thyrotropin worsens renal cortical perfusion and renal function in patients after total thyroidectomy due to differentiated thyroid cancer. Thyroid.

[16] Chaker L (2022). Hypothyroidism. Nat. Rev. Dis. Primers.

[17] Montenegro J (1996). Changes in renal function in primary hypothyroidism. Am. J. Kidney Dis..

[18] Kimmel M, Braun N, Alscher MD (2012). Influence of thyroid function on different kidney function tests. Kidney Blood Press. Res..

[19] Jayagopal V, Keevil BG, Atkin SL, Jennings PE, Kilpatrick ES (2003). Paradoxical changes in cystatin C and serum creatinine in patients with hypo- and hyperthyroidism. Clin. Chem..

[20] Hosten AO, Walker HK, Hall WD, Hurst JW (1990). BUN and Creatinine. Clinical Methods: The History, Physical, and Laboratory Examinations.

[21] Kumari B (2017). Serum BUN and creatinine estimation in patients of overt hypothyroidism: A case control study. Int. J. Res. Med. Sci..

[22] Beier K (2011). Elevation of blood urea nitrogen is predictive of long-term mortality in critically ill patients independent of “normal” creatinine. Crit. Care Med..

[23] Malik R, Hodgson H (2002). The relationship between the thyroid gland and the liver. QJM.

[24] Huang MJ, Liaw YF (1995). Clinical associations between thyroid and liver diseases. J. Gastroenterol. Hepatol..

[25] Targher G (2008). Association between serum TSH, free T4 and serum liver enzyme activities in a large cohort of unselected outpatients. Clin. Endocrinol. (Oxf.).

[26] Ajala MO, Ogunro PS, Fasanmade OA (2013). Relationship between liver function tests and thyroid hormones in thyroid disorders. Niger. Postgrad. Med. J..

[27] Levey AS (2006). Using standardized serum creatinine values in the modification of diet in renal disease study equation for estimating glomerular filtration rate. Ann. Intern. Med..

[28] Conover WJ (1999). Practical Nonparametric Statistics.

